# Estimate of nocturnal blood pressure and detection of non-dippers based on clinical or ambulatory monitoring in the inpatient setting

**DOI:** 10.1186/1471-2261-13-37

**Published:** 2013-05-21

**Authors:** Tan Xu, Yongqing Zhang, Xuerui Tan

**Affiliations:** 1 Department of Cardiology, the First Affiliated Hospital of Shantou University Medical College, Shantou, Guangdong 515041, China; 2 Department of Cardiology, Peoples’ Hospital of SanYan, SanYan, Hainan 572000, China

**Keywords:** Nocturnal blood pressure, Ambulatory blood pressure monitoring, Hypertension, Non-dipper

## Abstract

**Background:**

Ambulatory blood pressure monitoring is regarded as the gold standard for monitoring nocturnal blood pressure (NBP) and is usually performed out of office. Currently, a novel method for monitoring NBP is indispensible in the inpatient setting. The widely used manual BP monitoring procedure has the potential to monitor NBP in the hospital setting. The feasibility and accuracy of manual sphygmomanometer to monitor NBP has not been explored widely.

**Methods:**

A cross-sectional study was conducted at the cardiology department of a university-affiliated hospital to study patients with mild-to-moderate essential hypertension. One hundred and fifty-five patients were recruited to compare BP derived from a manual device and ambulatory BP monitoring (ABPM). The manual BP measurement was performed six times at 22:00, 02:00, 06:00, 10:00, 14:00 and 18:00 h. The measurements at 22:00, 02:00 and 06:00 h were defined as night-time and the others as daytime. ABPM was programmed to measure at 30-min intervals between measurements.

**Results:**

All-day, daytime and night-time BP did not differ significantly from 24-h ambulatory systolic BP [all-day mean difference −0.52±4.67 mmHg, 95% confidence interval (CI) –1.26 to 0.22, P=0.168; daytime mean difference 0.24±5.45 mmHg, 95% CI −0.62 to 1.11, P=0.580; night-time mean difference 0.30±7.22 mmHg, 95% CI −0.84 to 1.45, P=0.601) rather than diastolic BP. There was a strong correlation between clinical and ambulatory BP for both systolic and diastolic BP. On the basis of ABPM, 101 (65%) patients were classified as non-dippers, compared with 106 (68%) by manual sphygmomanometer (P<0.001).

**Conclusions:**

Traditional manual sphygmomanometer provides similar daytime and night-time systolic BP measurements in hospital. Moreover, the detection of non-dippers by manual methods is in good agreement with 24-h ABPM. Further studies are required to confirm the clinical relevance of these findings by comparing the association of NBP in the hospital ward assessed by manual monitoring with preclinical organ damage and cardiovascular and cerebrovascular outcomes.

## Background

In recent decades, although out-of-office blood pressure (BP) monitoring using ambulatory or home measurement techniques has evolved rapidly [1-3], clinical BP measurement has basically stayed the same [4-6]. Ambulatory BP monitoring (ABPM) has been regarded as the standard method for diagnosis and management of hypertension [7-9]. It is generally accepted that ABPM has better correlation with end-organ damage and cardiovascular outcomes compared with traditional office or clinic BP measurement techniques [10-13].

However, in clinical practice, particularly in hospital wards, BP mainly derived from traditional office blood pressure measurement is one of the most important vital signs [14]. In this context, comprehensive and accurate measurement of clinical BP (CBP) level is of utmost importance in the management of abnormal BP [15,16]. With regard to hypertension, appropriate assessment of CBP level is the premise for risk stratification and adequate therapeutic strategy. In clinical practice and research, CBP is usually just performed in the daytime. In the hospital ward, unless there are acute or severe conditions, CBP is rarely performed at night-time. Information about nocturnal BP (NBP) is extremely poor. Nevertheless, evidence has accumulated that NBP levels are more favorable in predicting the mortality and morbidity related to BP independently of daytime BP [17,18]. In contrast, for the management of BP morning surge, NBP is also indispensible [19]. Therefore, NBP should be taken seriously in the hospital setting for every patient, especially for those with abnormal BP.

Unfortunately, ABPM is regarded as the gold standard for monitoring NBP and is usually performed out of office [20]. Currently, a novel method for monitoring NBP is indispensible in the hospital setting. Probably the widely used manual BP monitoring has the potential to monitor NBP in the inpatient setting.

To date, research exploring the feasibility and accuracy of manual sphygmomanometer to monitor NBP has been rare. The present study compared 24-h ABPM with traditional manual techniques and used standard sphygmomanometer for the assessment of NBP and non-dippers among patients with essential hypertension in a ward setting.

## Methods

### Study design and population

This was a cross-sectional study in adults with essential hypertension who were attending the cardiology department of a university-affiliated hospital. The chief complaint of all recruited patients was dizziness, and they attended the hospital to establish the reasons for this dizziness. None of the participants had any acute lesions that influenced BP. Diagnosis and stratification of hypertension were based on recent guidelines [21]. Newly diagnosed patients or those on stable antihypertensive treatment for at least 4 weeks were recruited. During the monitoring period, the antihypertensive schedule had to remain the same as before.

Exclusion criteria were severe cardiac, renal or other systemic diseases, insomnia, and arrhythmia that interfere with ABPM devices, as well as patients with morbid obesity (body mass index >40 kg/m^2^). To ensure that evaluation of NBP was as accurate as possible, shift workers were also excluded. Secondary hypertension was ruled out using the procedure suggested in the international literature [22]. The Ethics Committee of the First Affiliated Hospital of Shantou University Medical College approved the study. All of the participants gave written informed consent.

### BP measurement and definitions

Daytime and night-time BP was measured by ABPM and manual monitoring in random order within 2 days in every patient. A standard sphygmomanometer (A&D UM-101, A&D, Tokyo, Japan) was used to measure daytime and night-time CBP. In the ward, all recruited patients were instructed to go to bed around 22:00 h and arise around 07:00 h. Six manual CBP measurements were obtained from 22:00 h at 4-h intervals between measurements. The actual times of CBP measurement were around 22:00, 02:00, 06:00, 10:00, 14:00 and 18:00 h. All the measurements were carried out by the same trained examiner. Before the monitoring, all patients were informed of the process and during the monitoring none of the participants were deliberately awakened. The desk lamp was switched on for the examiner to identify and write down the readings. Triplicate BP monitoring with at least 1 min between measurements using the same device was performed at the daytime points (10:00, 14:00 and 18:00 h) after the patients had refrained from exercise, coffee drinking, and smoking, and had relaxed for at least 5 min sitting in a quiet environment, as were the night-time measurements (22:00, 02:00 and 06:00 h) in the supine position. The inflatable cuff was always placed on the non-dominant upper limb. The mean of the triplicate BP readings was considered for analysis. Two inflatable bladder sizes were available according to the individual’s arm circumference (22 × 12 and 30 × 14 cm).

Ambulatory BP was monitored using validated oscillometric automatic-measuring device (A&D 2430) [23]. The ABPM device inflatable cuff was also placed on the non-dominant upper limb. The monitors were programmed to measure BP at 30-min intervals for 24 h on one of the two scheduled days. Patients were instructed to follow similar activities and diets as for the manual CBP measurement day, including no smoking or tea or coffee consumption. In addition, patients were advised to follow the guidelines of the monitors to avoid strenuous physical activities and to remain with the forearm extended during each ABPM session. When the ambulatory monitoring was completed, all the data were downloaded though a computer link using special software provided by A&D for analysis. Erroneous, valid and duplicate readings were flagged by the software. The daytime was fixed between 07:00 and 22:00 h because a fixed timetable for sleeping and arising had been scheduled for patients in the ward.

To guarantee that there was no consistent difference of ≥10 mmHg in the measured BP between ABPM devices and manual standard sphygmomanometer, before and after each CBP measurement or ABPM session, the accuracy of the monitors was assessed against a standard mercury sphygmomanometer (three successive readings; Y connector).

### Data analysis

CBP values with systolic BP (SBP) <90 mmHg or >200 mmHg and those with diastolic BP (DBP) <40 mmHg or >110 mmHg were considered erroneous and were discarded. If any of the aforementioned six consecutive BP readings was invalidated or the sleeping duration was <4 h, that patient’s data were excluded from the following analysis. Three daytime (10:00, 14:00 and 18:00 h) and three night-time (22:00, 02:00 and 06:00 h) CBP readings were averaged to give a single daytime and night-time CBP value per patient. Dipping pattern was defined as follows: dippers had a ≥10% but <20% fall in NBP; non-dippers had an NBP fall <10% but >0%; extreme dippers had an NBP fall ≥20%; and reverse dippers had an NBP increase [24].

The 24-h ABPM data were divided into daytime (07:00–22:00 h) and night-time (22:00–07:00 h) values. ABPM measurements with three consecutive or a total of five invalidated readings were regarded as erroneous and were discarded, as were measurements with SBP <60 mmHg or >250 mmHg and those with DBP <30 mm Hg or >150 mmHg. Erroneous readings and duplicate readings that the software flagged were also discarded. The means of all valid daytime and night-time ABPM readings were to give a single daytime and night-time ABPM value per patient.

The data were stored and analyzed using the MS Excel program. The statistical analysis was carried out using SPSS for Windows release 13.0. The *t* test for paired samples was used to compare BP measurements in the same individual and χ^2^ test was used to compare the categorical variables. Pearson correlation coefficient and intraclass correlation coefficient were used to estimate the association between continuous variables. The κ statistic was used to determine the level of agreement in diagnosis of non-dippers measured by ambulatory blood pressure (ABP) and CBP measurements. Two-tailed values of P<0.05 were considered statistically significant.

## Results

### Patient characteristics

A total of 169 patients were recruited and 14 were excluded because of inadequate ABPM data. Therefore, 155 patients were included in the final analysis (newly diagnosed with essential hypertension 44.52%, men 51%, smokers 23.9%, mean age 67±12 (SD) years, body mass index 23.53±2.92 kg/m^2^, CBP 160.79±23.19/90.74±14.46 mmHg, SBP/DBP). Twenty-seven (17.4%) patients had type 2 diabetes mellitus. Sixty-five participants (41.9%) had a history of stroke and 32 (20.6%) had a previous major cardiovascular disease. During the manual measurement of NBP, about one half of participants (46%) were awake.

### BP measurements

**Figure 1:**
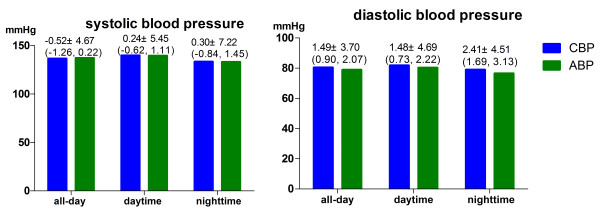
**Comparison of clinical blood pressure measured by manual device and ambulatory blood pressure measurements (mean difference ± SD; 95% confidence intervals in parentheses).** CBP, clinical blood pressure; ABP, ambulatory blood pressure.

All-day CBP did not differ significantly from 24-hour ABP for SBP [mean difference −0.52±4.67 mmHg, 95% confidence interval (CI) –1.26 to 0.22, P=0.168; Figure 1). This was also the case for daytime and night-time systolic CBP as compared with ABP (daytime mean difference 0.24±5.45 mmHg, 95% CI −0.62 to 1.11, P=0.580; night-time mean difference 0.30±7.22 mmHg, 95% CI −0.84 to 1.45, P=0.601; Figure 1). When compared with DBP, significant differences were observed between manual sphygmomanometer and ABPM for all-day (mean difference 1.49±3.70 mmHg, 95% CI 0.90–2.07, P<0.001), daytime (mean difference 1.48±4.69 mmHg, 95% CI 0.73–2.22, P<0.001), and night-time (mean difference 2.41±4.51 mmHg, 95% CI 1.69–3.13, P<0.001) (Figure 1). There was a strong correlation between CBP and ABP for both SBP and DBP (Table 1).

**Table 1:** Comparison of the correlation in BP measured by manual sphygmomanometer and ABPM

**CBP-ABP**	**r**	**P**	**ICC**	**95% IC**	**P**
All-day systolic	0.97	<0.001	0.944	0.924-0.959	<0.001
All-day diastolic	0.89	<0.001	0.878	0.836-0.969	<0.001
Daytime systolic	0.925	<0.001	0.922	0.895-0.943	<0.001
Daytime diastolic	0.831	<0.001	0.824	0.767-0.869	<0.001
Nighttime systolic	0.906	<0.001	0.903	0.869-0.928	<0.001
Nighttime diastolic	0.869	<0.001	0.857	0.809-0.894	<0.001

CBP: clinical blood pressure performed by manual device, ABP: ambulatory blood pressure, r: correlation coefficients r, ICC: intraclass correlation coefficients, CI: confidence interval.

### Detection of non-dippers

On the basis of ABPM, 101 (65%) patients were classified as non-dippers, as compared with 106 (68%) by manual sphygmomanometer (P<0.001). When taking ABPM as the reference standard to determine non-dippers, the agreement was 82.58% as compared with CBP (κ 0.608, P<0.001). Furthermore, when dividing the circadian rhythm of BP into four groups, 61 participants were classified as reverse-dippers (night-time BP higher than daytime) by ABPM and 62 by CBP (diagnostic agreement 74.84%, κ 0.474, P<0.001). Five patients were defined as extreme-dippers (nocturnal dip >20% for SBP/DBP) by ABPM and five by CBP (diagnostic agreement 96.13%, κ 0.380, P<0.001). When non-dippers were assessed separately for SBP and DBP, the diagnostic agreements between ABP and CBP are shown in Table 2.

**Table 2:** Diagnostic agreement between CBP and ABP in non-dippers

**CBP-ABP**	**Agreement (%)**	**Kappa**	**P**
Systolic	84.52	0.596	P<0.001
Diastolic	83.23	0.521	P<0.001
Systolic or Diastolic	82.58	0.608	P<0.001

CBP: clinical blood pressure performed by manual device, ABP: ambulatory blood pressure, the Kappa value was calculated in the condition of ABP as the golden standard.

## Discussion

In this study, we compared manual sphygmomanometer monitoring with 24-h ABPM in the inpatient setting, in terms of daytime and night-time BP levels and the detection of BP circadian rhythm. The main conclusions were that: (i) there were no differences between CBP and ABP measurements taking during the daytime and night-time for SBP rather than diastolic BP; and (ii) there was good agreement between CBP and ABP in the detection of non-dippers.

Since ABPM was firstly described >40 years ago, it has been generally accepted that it can provide the following three types of information: an estimate of mean BP level; diurnal rhythm of BP; and BP variability [20], particularly NBP. Currently, ABPM is considered as the gold standard for NBP measurement [20,25]. However, this seemingly perfect method for NBP measurement has its own limitations, for example, disturbing sleep quality [26], high cost, and inconvenience of performing multiple measurements. The feasibility of obtaining other NBP measurement methods has been investigated widely, especially using home-monitoring devices. In 2001, a newly developed self-measurement BP device (Omron HEM 747-IC-N, Omron Life Science, Tokyo, Japan) was investigated to monitor NBP at 02:00 h [27]. The conclusion was that the device could not provide ideal information on BP during sleep [27]. Six years later, the aforementioned self-measurement device was investigated to assess the reproducibility in two different nights at a mean 5.9 days apart in 556 participants [28]. This study concluded that the reproducibility was poor, especially in individuals who experienced good sleep quality in just one session [28].

A recent study in 40 healthy individuals has provided a direct comparison between night-time home BP monitoring and ABPM. No significant difference was found between the two methods for measurement of NBP [29]. Another more recent study [30] has compared ABP levels with home BP values in 81 patients with hypertension. NBP measurement was performed three times per night on three nights within 2 weeks using automated devices (Microlife WatchBPN; Microlife, Widnau, Switzerland). A strong correlation was found between ABP levels and home BP values [30]. This study concluded that home BP monitoring may be a reliable alternative to ABP for the assessment of NBP and the detection of non-dippers [30].

To the best of our knowledge, no studies have assessed the potential of manual sphygmomanometer devices to monitor NBP in an inpatient setting. Also, no studies have directly compared NBP obtained by manual sphygmomanometer monitoring and ABPM. BP is one of the vital signs and in hospital wards, it must be taken seriously. Isolated nocturnal hypertension upon ambulatory measurement as a novel clinical entity was first described by Li et al. [31]. Isolated nocturnal hypertension predicts cardiovascular outcome in patients who are normotensive in the office or with ambulatory daytime BP measurement [32]. Nonetheless, in clinical settings, unless there are acute or severe conditions, NBP has not received enough attention. In contrast, NBP measurement is also required to determine the cause of the morning surge in BP. The indispensible importance of NBP has been widely accepted. Therefore, we need to acknowledge the importance of NBP not only out of office but also in clinical settings, regardless of the condition of the patients.

In the present study, we compared CBP measured by manual devices with automated ABPM. Regardless of the all-day, daytime or night-time measurements, no significant difference was found between the CBP and ABP for SBP, despite a small number of readings being obtained per night (*n*=3). The current study showed that NBP monitoring performed by manual sphygmomanometer gave similar values compared with ABPM. In contrast, strong correlations were found between CBP and ABP. Despite there being a small difference between ABP and CBP in monitoring of NBP, the two methods appeared to be interchangeable in the detection of NBP in the clinical setting. In our study, the two methods differed significantly between CBP and ABP for DBP. The divergence of SBP and DBP needs further study. Several reasons may explain the divergence. First, manual monitoring or ABPM influence DBP more than SBP. Second, sleep quality may be affected more by DBP than SBP. Finally, there is probably no correlation between night-time DBP using the two methods.

The most challenging finding of the present study was that there was good agreement in the detection of non-dippers, and moderate and fair agreement for reverse dippers and extreme dippers, respectively. In 155 patients with hypertension, >50% were found to be non-dippers using both methods. Furthermore, about 60 patients were classified as reverse-dippers. This finding warned us that in the clinical setting, nocturnal dipping of BP is much less frequent, and even the NBP is much higher than the daytime BP. It may have been that the patients were under stress, and the percentage of non-dippers was high. Therefore, we should not ignore the NBP in the hospital setting. It could be that the ideal management of BP in the daytime results in abnormal BP at night. The present study showed that traditional widely used manual devices for monitoring BP may give reliable NBP measurement compared with ABPM. It should be noted, however, that there are no other available data to define the non-dippers in clinical settings, regardless of ABPM or other methods. More studies should be performed to investigate the relationship between non-dippers measured by ABPM or CBP monitoring in hospital wards and target organ damage or mortality.

### Study limitation

The present study must be interpreted within the context of its potential limitations. First, BP measurement was only performed on 2 days, and one type of method was just implemented once. Therefore, the reproducibility could not be analyzed. Second, this study did not evaluate the sleep quality associated with the two methods, which may have seriously influenced NBP. Third, the study fixed the daytime and night-time using an arbitrary range, which may have affected the definition of non-dippers.

## Conclusion

Our data suggest that traditional manual sphygmomanometer provides similar daytime and night-time SBP in the hospital ward. Moreover, the detection of non-dippers by the manual methods is in good agreement with 24-h ABPM, but moderate and fair agreement for reverse dippers and extreme dippers, respectively. One challenge of the study is that more than half of the patients in the ward had high NBP. Further studies are required to confirm the clinical relevance of these findings by comparing the association of NBP in the hospital ward assessed by manual sphygmomanometer monitoring and ABPM with preclinical organ damage and cardiovascular and cerebrovascular outcomes.

## Competing interests

1. In the past five years all the authors haven’t received any reimbursements, fees, funding, or salary from an organization that may in any way gain or lose financially from the publication of this manuscript. 2. All the authors don’t hold any stocks or shares in an organization that may in any way gain or lose financially from the publication of this manuscript. 3. All the authors don’t hold or currently apply for any patents relating to the content of the manuscript. 4. All the authors don’t have any other financial competing interests. 5. There is a non-financial competing interests related to this manuscript, which I have declared in the acknowledgment. This work was supported by the Provincial Natural Science Foundation of Guangdong.

## Authors’ contributions

TX performed the survey and wrote the draft of manuscript. YQZ supervised data collection. XRT designed the study, helped interpret the analysis results, and assisted in drafting and modifying the manuscript. All authors read and approved the final manuscript.

## Pre-publication history

The pre-publication history for this paper can be accessed here:

http://www.biomedcentral.com/1471-2261/13/37/prepub
